# Phase Transitions in Chemically Fueled, Multiphase Complex Coacervate Droplets

**DOI:** 10.1002/anie.202211905

**Published:** 2022-10-18

**Authors:** Carsten Donau, Fabian Späth, Michele Stasi, Alexander M. Bergmann, Job Boekhoven

**Affiliations:** ^1^ Department of Chemistry Technical University of Munich Lichtenbergstrasse 4 85748 Garching Germany

**Keywords:** Chemically Fueled, Complex Coacervation, Membraneless Organelles, Multiphase Droplets, Phase Transitions

## Abstract

Membraneless organelles are droplets in the cytosol that are regulated by chemical reactions. Increasing studies suggest that they are internally organized. However, how these subcompartments are regulated remains elusive. Herein, we describe a complex coacervate‐based model composed of two polyanions and a short peptide. With a chemical reaction cycle, we control the affinity of the peptide for the polyelectrolytes leading to distinct regimes inside the phase diagram. We study the transitions from one regime to another and identify new transitions that can only occur under kinetic control. Finally, we show that the chemical reaction cycle controls the liquidity of the droplets offering insights into how active processes inside cells play an important role in tuning the liquid state of membraneless organelles. Our work demonstrates that not only thermodynamic properties but also kinetics should be considered in the organization of multiple phases in droplets.

## Graphical abstract

Coupling multiphase coacervate droplets to a chemical reaction cycle allows access to regimes inside the phase diagram not possible under thermodynamic control. Control over the stability, liquidity, and localization of the multiphases makes it a great synthetic platform to study the organization of membraneless organelles.

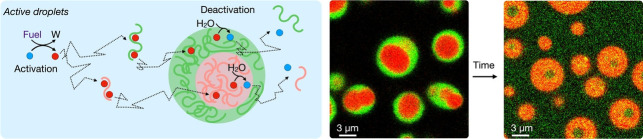

## Introduction

Membraneless organelles have emerged as a major part of the intracellular organization.[^1^, ^2^, ^3^] They assemble through liquid‐liquid phase separation of a dense protein and RNA‐rich phase in the cytosol where they perform a plethora of cellular functions like modulating enzymatic activity, buffering noise in protein concentrations, or influencing signaling pathways.[^4^, ^5^] Increasing evidence suggests that some membraneless organelles are not homogeneous but instead have subcompartments that further separate components through different partitioning between the phases.[^6^, ^7^, ^8^, ^9^, ^10^, ^11^] Furthermore, many membraneless organelles are active compartments that are regulated by chemical reactions, i.e., they are kinetically controlled under conditions away from equilibrium.[^5^, ^12^, ^13^, ^14^, ^15^, ^16^, ^17^, ^18^, ^19^, ^20^, ^21^, ^22^] Chemical reactions may change their liquid‐like property[^3^, ^5^, ^15^, ^23^, ^24^] and malfunction has been linked to disease.[^25^, ^26^, ^27^, ^28^] However, how exactly chemical reactions regulate membraneless organelles, and the connection between molecular design and their emergent properties remains elusive.[^2^, ^13^, ^17^, ^25^]

Synthetic coacervate droplets have proven to be a powerful model for membraneless organelles.[^29^, ^30^, ^31^, ^32^, ^33^, ^34^] These droplets form through attractive interactions between polymers via ion‐pairing and other supramolecular effects to give membraneless droplets with liquid‐like properties.[^33^, ^35^] Recently, it was shown that coacervate droplets with subcompartments can also be formed[^36^, ^37^, ^38^, ^39^, ^40^, ^41^] which rely on differences in surface tension between the respective phases,^[37]^ similar to membraneless organelles.^[6]^ However, kinetically controlled multiphase droplets are rare.^[42]^ Such models are powerful to explore mechanisms by which chemical reactions regulate phase transitions, internal droplet hierarchy, and droplet properties. In this work, we thus set out to understand the mechanisms by which chemical reactions can regulate the internal hierarchy of multiphase coacervate droplets. We use a chemical reaction cycle that regulates the affinity of a peptide for polyelectrolytes as a model for post‐translational modifications, e.g., protein phosphorylation, acetylation, or methylation, to regulate their ability to phase separate. By combining the peptide with two polyelectrolytes we create a system without droplets at equilibrium, or where droplets are formed with or without sub‐compartmentalization depending on how far the system is brought out of equilibrium. We study in detail the transitions from one regime to another and identify new transition states that are not observed under thermodynamic control. Finally, we show that the chemical reaction cycle controls the liquidity of the droplets yielding insights into how active processes inside cells play an important role in tuning the liquid state of membraneless organelles. Our work demonstrates that not only thermodynamic properties but also kinetics should be considered in the organization of multiple phases in droplets.

## Results and Discussion

**Figure 1:**
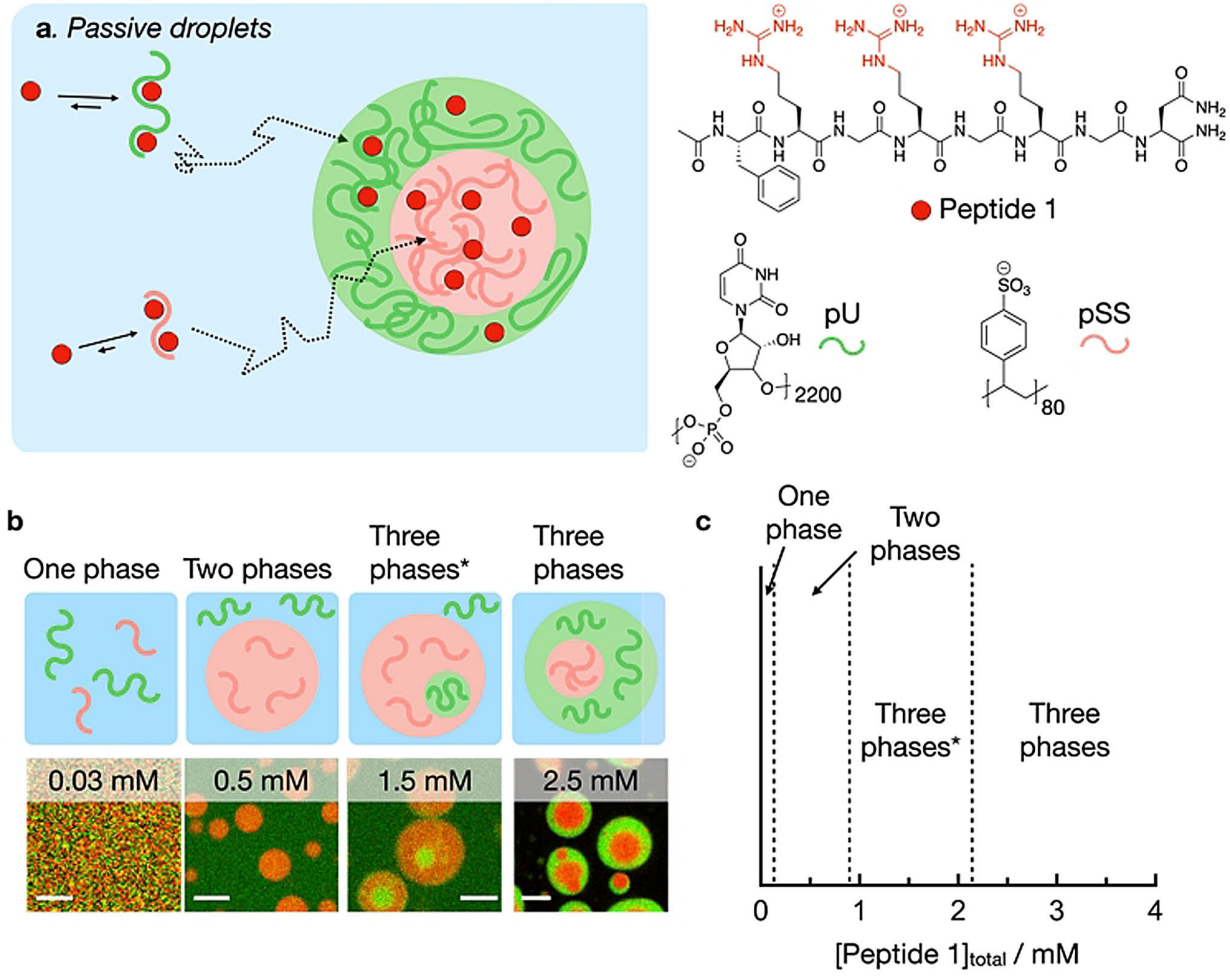
Multiphase droplets under thermodynamic control. a) Schematic representation of the phase separation of pU, pSS, and peptide 1. b) Schemes and respective confocal micrographs of droplets from solutions containing various concentrations of peptide 1 and 5 mM pU (expressed as monomer units), 5 mM pSS (expressed as monomer units) in 200 mM MES at pH 5.3 with 0.15 μM Cy3‐A_15_‐pU (green) and 0.15 μM Cy5‐pSS (red), 10 minutes after droplet formation. Scale bar: 3 μm. c) Phase diagram as a function of the total concentration of peptide 1 of solutions from (b).

To design droplets with subcompartments (multiphase droplets) regulated by chemical reactions, we first screened for the conditions that resulted in multiphase droplets close to equilibrium, i.e., droplets that are not actively maintained by chemical reactions. We refer to these as passive droplets (Figure 1a). We used a small cationic peptide (peptide 1) and combined it with two polyanions, i.e., poly‐U (pU, 600–1000 kDa) and polystyrene sulfonate (pSS, 17 kDa). Peptide 1 has the following structure: Ac‐FRGRGRGN‐NH_2_ and it contains 1) F, i.e., phenylalanine important for aromatic interaction with the polyanions, 2) the RG repeat, i.e., arginine‐glycine repeat that is known to bind polyanions and 3) an amidated N, i.e., asparagine. We chose the asparagine for this peptide, because, when mutated for aspartic acid, it makes the peptide responsive to a chemical reaction cycle which we explain below.[^32^, ^43^, ^44^]

We established the interaction strength of peptide 1 with pSS and pU individually in 200 mM MES buffer at pH 5.3 which are the conditions used throughout his work. At this pH, peptide 1 has an overall charge of +3. Isothermal titration calorimetry (ITC) showed that peptide 1 has a higher affinity for pSS compared to pU (*K*_D_ of 3.2 μM vs 130 μM, Figure S1). This result was further corroborated by turbidity measurements that we carried out using a UV/Vis spectrophotometer at 600 nm (Table S1) which showed the appearance of turbidity above a critical coacervation concentration (CCC) of 0.8 mM of peptide 1 combined with pSS (10 mM), and 1.85 mM with pU (10 mM). We also used turbidity measurements to determine the critical salt concentration, i.e., the amount of salt added required to dissolve the droplets. 1.5 M NaCl was not enough to revert to a clear solution for pSS (10 mM pSS, 2.5 mM peptide 1), while 63 mM NaCl was sufficient in the case of pU under similar conditions (Table S1). This critical salt concentration is an indirect measure of the interfacial surface tension between the droplet phase and buffer,^[37]^ and a large difference in this value is the driving force for multiphase droplets to form. It is therefore likely that peptide 1 will form multiphase droplets when combined with both pSS and pU.

We confirmed the presence of multiphase droplets by confocal microscopy. We used a solution of pU, pSS, peptide 1, and peptide 2. Peptide 2 is structurally very similar to peptide 1 with the amino acid sequence: Ac‐FRGRGRGD‐OH. The aspartic acid at the C‐terminus is negatively charged, resulting in a zwitterionic peptide with an overall charge of +1. ITC experiments showed that peptide 2 has two orders of magnitude lower affinity for the two polyanions than peptide 1 (Figure S1). Thus, peptide 2 serves mostly as a molecular crowder, but we used it in later experiments to compare passive droplets with fuel‐driven droplets. We ensured that the total peptide concentration was always 15 mM, i.e., [peptide 1]+[peptide 2]=15 mM. More than 0.05 mM of peptide 1 was needed to form droplets (Figure 1b and c). The addition of 0.5 mM of peptide 1 led to the formation of droplets with a diameter of a couple of micrometers that contained pSS (red color) but did not up‐concentrate pU (green). Above 0.9 mM peptide 1, pU droplets started to form which settled inside the larger pSS droplets. With the addition of more peptide 1, the pU droplets grew larger and we observed multiphase droplets with a pSS core surrounded by a pU shell (2.2 mM peptide 1 or higher). This result is in line with the CSC experiments where we showed that the pSS‐buffer interface has a higher surface tension compared to the pU‐buffer interface. Energetically speaking, it is thus favorable to settle the pSS droplets into the core of the multiphase droplets. Between 0.9 mM and 2.2 mM peptide 1, pU droplets are not sufficiently large to surround the pSS droplets. In this case, multiphase droplets are energetically favorable where the shell is composed of the phase that has the highest interfacial surface energy with the buffer phase (Three phases*, Figure 1c).^[37]^ Confocal micrographs with the fluorescent analog of peptide 1 showed that peptide 1 preferentially partitions into the pSS core (Figure S2) in accordance with the ITC measurements. Control experiments showed that peptide 1 partitions similarly to its fluorescent analog (Figure S3). Taken together, the peptide 1 concentration regulates whether one, two, or three phases are obtained.

**Figure 2:**
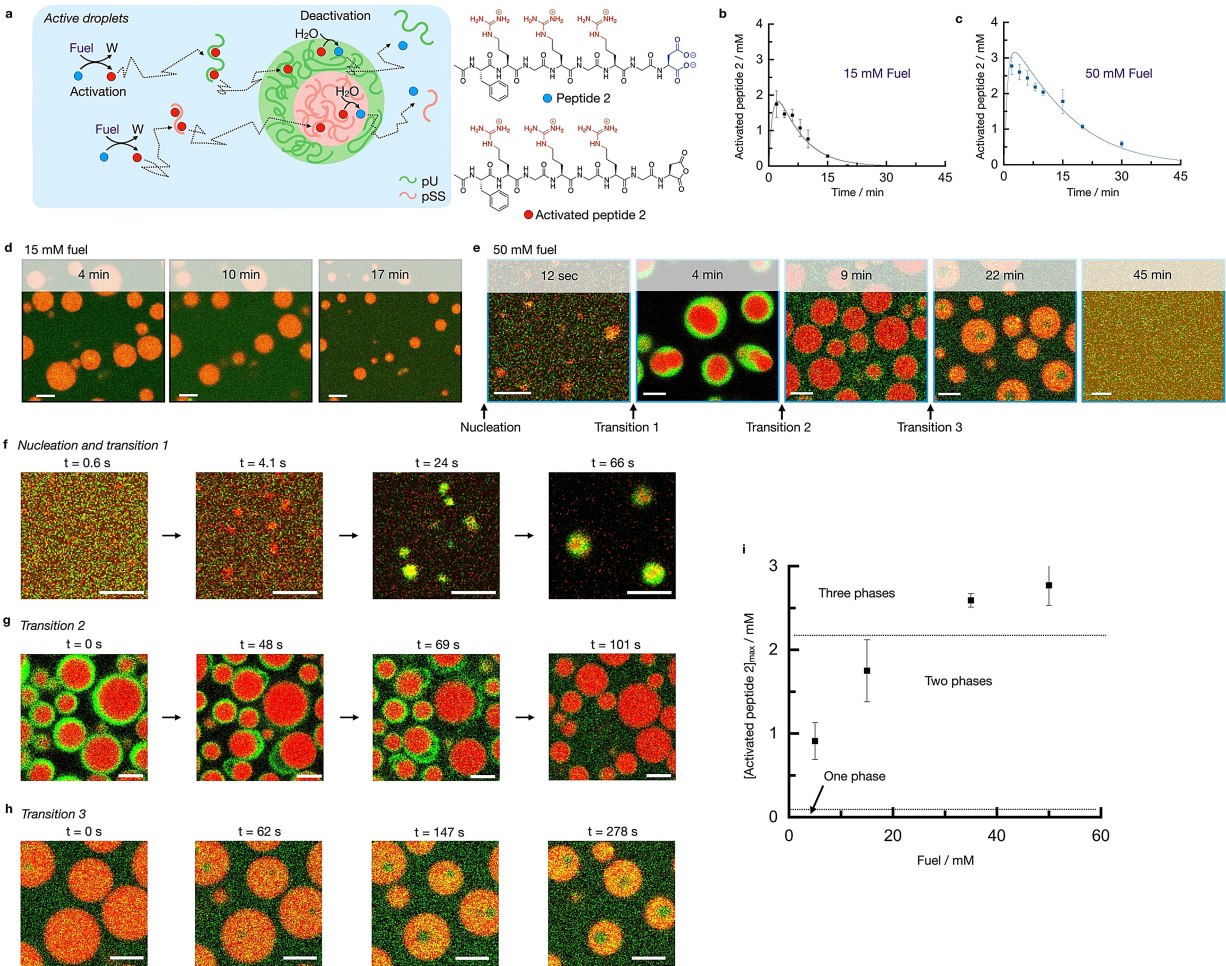
Phase transitions in active multiphase droplets under kinetic control. a) Schematic representation of the kinetically controlled phase separation of pU, pSS, and peptide 2. The conversion of fuel into a waste product (W) activates peptide 2 for phase separation. Activated peptide spontaneously deactivates upon reaction with water. In its finite lifetime, it complexes pU or pSS and phase separates into multiphase droplets. b–c) Concentration of activated peptide 2 as a function of time for 15 mM (b) and 50 mM (c) fuel. Solutions containing 15 mM peptide 2, 5 mM pU, 5 mM pSS, 200 mM MES at pH 5.3. The solid lines represent calculated trends by our kinetic model. d–e) Confocal micrograph time series of solutions from (b) or (c) with 0.15 μM Cy3‐A_15_‐pU (green) and 0.15 μM Cy5‐pSS (red). f) Confocal micrographs of the nucleation and transition 1 of solutions from (c), right after fuel addition. g–h) Confocal micrographs of transitions 2 (g) and 3 (h) of solutions from (c). i) Maximum concentration of activated peptide 2 after 2 minutes as a function of fuel concentration. Dotted lines indicate at which activated peptide 2 concentrations two or three phases are obtained. All error bars show the standard deviation from the average (N=3). Scale bar for all micrographs: 3 μm.

To test how chemical reactions can regulate these multiphase droplets, we converted the passive, multiphase droplets into active ones. Thus, we used peptide 2 as a precursor in our previously described fuel‐driven reaction cycle.[^32^, ^43^, ^45^] Peptide 2 alone cannot form droplets with pSS or pU under the employed conditions. However, the chemical reaction cycle can temporarily activate the peptide for droplet formation at the expense of a chemical fuel (Figure 2a, Figure S4). Peptide 2 (Ac‐FRGRGRGD‐OH) can react with 1‐ethyl‐3‐(3‐dimethylaminopropyl) carbodiimide (fuel) to convert its C‐terminal aspartic acid into its cyclic anhydride state. That activated state, which we refer to as activated peptide 2, has a short half‐life time of about 45 seconds before it is hydrolyzed to revert to the original peptide 2, i.e., the deactivation reaction in the cycle. Activated peptide 2, due to the loss of its two anionic carboxylates has an overall charge of +3 and is therefore expected to behave like peptide 1 in the above‐described passive droplets, i.e., have a high affinity for the polyanions and form multiphase droplets.

The amount of activated peptide 2 generated and for how long it remains present can be controlled by the amount of fuel added. We measured the concentrations of activated peptide 2 and fuel over time by HPLC (Figure 2b and c, Figure S5) and used that data to adjust a previously written kinetic model.[^32^, ^46^] The kinetic model predicts the concentrations of all reactants and products every second after fuel is added and can be used to predict the maximum concentration of activated peptide 2 reached. For example, when 15 mM of fuel was added, the concentration of activated peptide 2 rapidly increased to just below 2 mM after which it decayed back to 0 mM within 20 minutes (Figure 2b). In contrast, when 50 mM fuel was added, the concentration of activated peptide 2 rapidly increased to just below 3 mM and decayed over the next 40 minutes (Figure 2c). Turbidity measurements showed that the droplet formation is following the evolution of activated peptide 2 (Figure S6).

Using confocal microscopy, we found droplets that contained pSS in the case of 15 mM fuel (Figure 2d). These droplets emerged almost immediately, grew via fusion, and dissolved after 20 minutes (Movie S1). Under those conditions, no evidence of multiphase droplets was found. In contrast, in the case of 50 mM fuel, multilayered droplets were obtained with a pSS core and a pU shell in the first minutes (Figure 2e). Strikingly, we were able to resolve the nucleation of the pSS core and the pU shell by employing a microfluidic setup that mixes and entraps all ingredients for the complex coacervate droplets in a 40 μm‐sized water droplet (Movie S2). While the pSS phase formed within a second after fuel addition, the pU shell (droplet transition 1) formed after just over 20 seconds (Figure 2f, Movie S3). We also used confocal fluorescence microscopy in the bulk solution for 50 mM fuel. After 9 minutes, we observed a droplet transition in which multiphase droplets lost their pU shell (droplet transition 2, Figure 2e). The time of the transition coincides with the concentration activated peptide 2 falling below 2 mM. The transition does not occur smoothly through the dissolution of the shell but instead, the pU phase swells and detaches from the pSS phase followed by the division of the pU phase into fragments of pU (Figure 2g, Movie S4). The fragments of the pU‐based shell survive for several seconds before they eventually also dissolve.

We explain the swelling and the detaching of the pU phase by the increase in negative charges due to the continuous hydrolysis of activated peptide 2. This observation is in line with our previous work.^[32]^ We captured the bursting of the pU shell in microfluidics as well (Movie S5).

Surprisingly, between 9 and 20 minutes, we found evidence of another unexpected transition. Where we expected the pSS droplets to dissolve, we found that the pSS droplets had suddenly obtained a pU core (droplet transition 3, Figure 2e). The pU that previously made up the shell had dissolved and reenters the droplet to form a new droplet core (Figure 2h, Movie S6). Noteworthy, we excluded that this transition is a consequence of the dehybridization of Cy3‐A_15_ from pU (Figure S7) (see supplementary discussion 1). This process was accompanied by droplet shrinkage until the droplets finally dissolved after more than 40 minutes. We also observed this transition in the microfluidic setup (Figure S8). This transition is surprising because the pU phase should not be stable under these concentrations of activated peptide 2 below 1 mM (see passive droplets). The observation suggests that pU plays a dual role, both as the shell and the core and its location can be regulated kinetically by the balance between activation and deactivation.

Based on the confocal microscopy data and the kinetic model, we establish a phase diagram to explain the three phase transitions (Figure 2i). We found that 0.7 mM of fuel was needed to observe pSS droplets. According to the kinetic model, that amount of fuel results in a maximum concentration of activated peptide 2 of 0.1 mM, i.e., activated peptide 2 has a CCC of 0.1 mM to form droplets with pSS, similarly to peptide 1 in passive droplets. When the concentration of activated peptide 2 was above 2.0 mM, we found multiphase droplets with a pU shell. Thus around 2.0 mM, there is a CCC for the formation of the pU shell, similarly to peptide 1 in the passive droplets. These results indicate that active droplets behave similarly to the passive droplets formed by peptide 1 in terms of their phase diagram. However, the surprising formation of a pU core at the end of the reaction cycle (transition 3) is not explained by the phase diagram of passive or active droplets.

**Figure 3:**
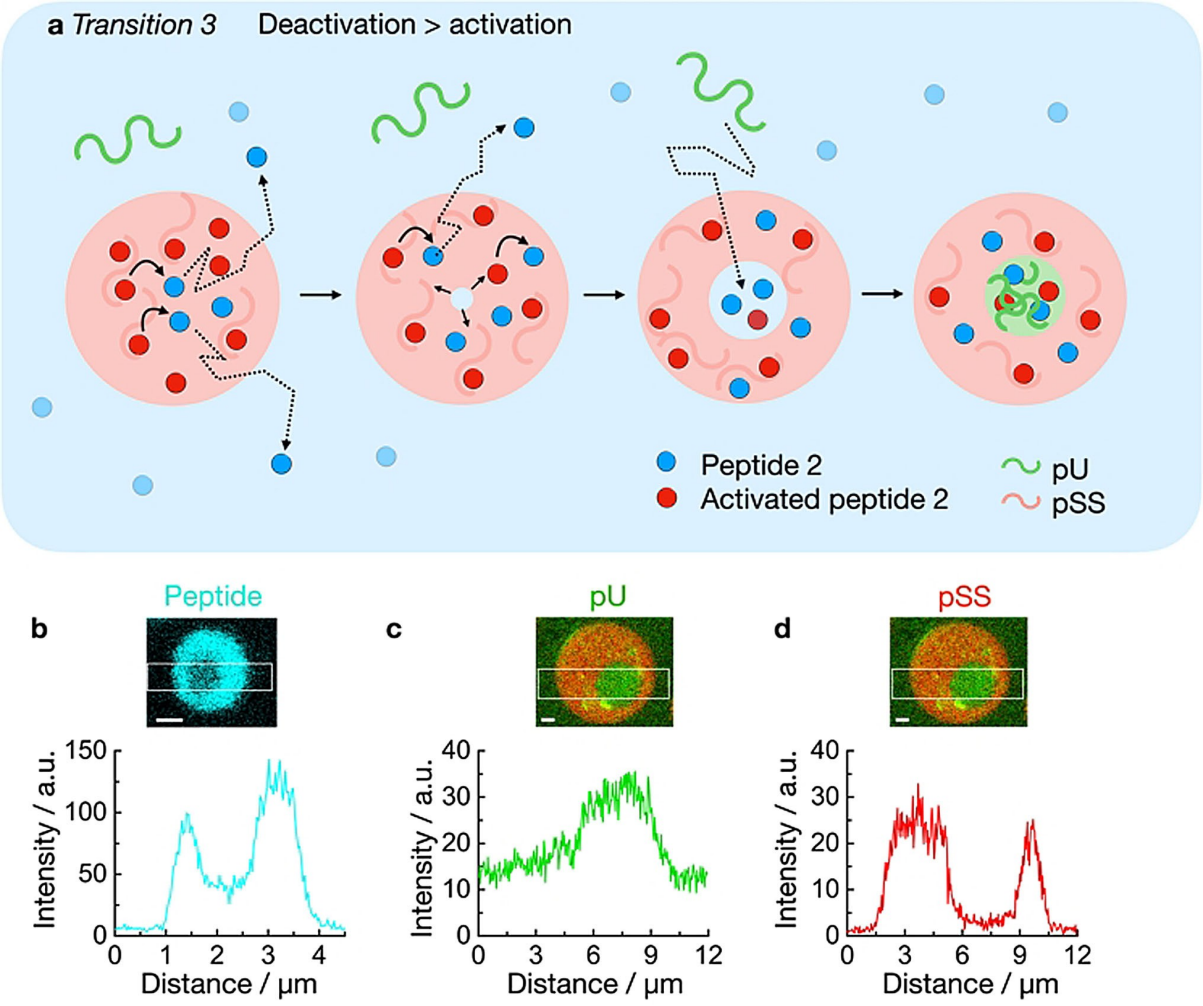
Mechanism of transition 3 in active droplets. a) Schematic representation of the vacuole formation of an active droplet containing pSS in presence of pU. Peptide deactivation leads to the build‐up of osmotic pressure and the nucleation of a dilute phase inside the droplet which harbors elevated peptide concentrations compared to the surrounding dilute phase. The influx of pU leads to the formation of a new coacervate phase. b) Plot profile of peptide in a droplet, 20 minutes after addition of 50 mM fuel. Solutions containing 15 mM peptide 2, 5 mM pU, 5 mM pSS, 200 mM MES, pH 5.3, 1 μM NBD‐GRGRGRGD‐OH (blue). c–d) Plot profile of pU (c) and pSS (d) in a droplet, 20 minutes after addition of 50 mM fuel. Solutions from (b) but with 0.15 μM Cy3‐A_15_‐pU and 0.15 μM Cy5‐pSS (red) instead of NBD‐peptide. Scale bar: 1 μm.

To explain transition 3, we propose that individual pU molecules diffuse into the core of the pSS droplet (Figure 3a). Indeed, we found pU in the pSS phase (Figure 3c) which shows fast recovery after photobleaching (FRAP, Figure S9). We propose that the driving force for pU to enter and form the multiphase core is vacuole formation. Vacuole formation in coacervate droplets is the process in which a droplet transitions into an aqueous phase surrounded by phase‐separated material. Vacuolization has been linked to the build‐up of osmotic pressure within the droplet and is frequently observed in droplet dissolution.[^32^, ^47^, ^48^, ^49^, ^50^] Thus the aqueous environment of the vacuole has a higher concentration of charges compared to the dilute phase. The increased charge serves as a driving force for the up‐concentration of pU.

In our active droplets, activated peptide 2 is constantly deactivated within the droplets (Figure 3a). As the system is running low on chemical fuel, the activation that supplies the droplet with activated peptide 2 is outcompeted by the deactivation leading to droplet dissolution. As a result, a vacuole forms. We confirmed that the vacuole contains a higher concentration of peptide than the dilute phase (Figure 3b). That increased concentration of peptide attracts pU into the core of the droplet. Indeed, pU was also up‐concentrated in the core compared to the phase outside of the droplets (K=3, Figure 3c) whereas the concentration of pSS was the highest in the droplet phase surrounding the vacuole (Figure 3d). Specifically, the concentration of peptide was about 89 mM in the vacuole (see methods, Table S2). We validated that 89 mM peptide 2 can create multiphase droplets even without fuel which further indicates that the vacuole is a new phase vastly different from the dilute phase outside of the droplets (Figure S10).

Noteworthy, experiments without pU showed that vacuolization is still part of the dissolution process of pSS droplets with 50 mM fuel (Movie S7). In other words, pU is not required for vacuolization. In contrast, the same experiment with only 15 mM fuel did not show the formation of vacuoles (Movie S8). In line with that, 15 mM fuel with pU did not show the formation of a pU core (see Figure 2d), which strongly indicates that vacuolization is required for transition 3.

Taken together, vacuolization is part of the dissolution process of our droplets when the system is running low on fuel. The concentration of peptide in the vacuole is vastly higher compared to the dilute phase which attracts pU (transition 3). This new phase is kinetically stabilized by the surrounding shell which is not observed for passive droplets due to the absence of a deactivation reaction. Coupling the droplet properties to a chemical reaction cycle thus allows for access to regimes in the phase diagram not possible under thermodynamic control. Excitingly, increasing studies suggest that vacuole formation occurs in membraneless organelles and related protein droplets, too.[^51^, ^52^, ^53^, ^54^, ^55^, ^56^, ^57^]

**Figure 4:**
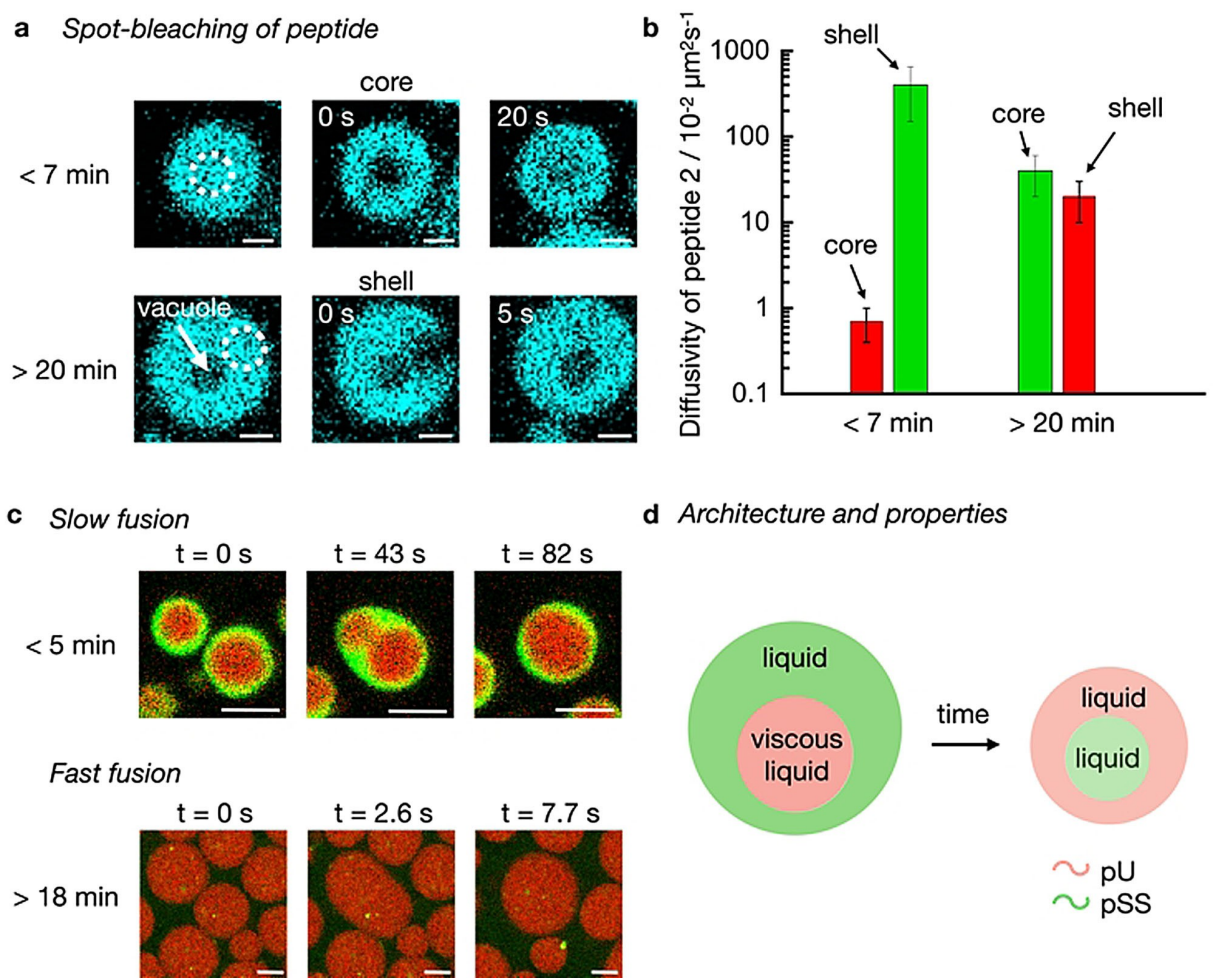
Diffusivity of molecules in active droplets. a) Confocal micrographs from FRAP spot bleaching experiments less than 7 or more than 20 minutes after the addition of fuel. The solutions contain 15 mM peptide 2, 5 mM pU, 5 mM pSS, 200 mM MES, pH 5.3, 1 μM NBD‐GRGRGRGD‐OH (cyan) with 50 mM fuel. Scale bar: 1 μm. b) Diffusivity of peptide 2 (and activated peptide 2) in the core and the shell of active multiphase droplets from (a) as a function of time. The colors indicate the pU (green) and pSS (red) phases. Error bars show the standard deviation of 6 droplets from 2 experiments (N=6). c) Confocal micrographs of fusion events from solutions of (a) with 0.15 μM Cy3‐A_15_‐pU (green) and 0.15 μM Cy5‐pSS (red). Scale bar: 2 μm. d) Schematic representation of active droplets with inner hierarchy. Properties of inner and outer layers change over time.

Membraneless organelles’ subcompartments vary in chemical composition, but also vary in viscosity, diffusivity, and reactivity.^[58]^ We performed FRAP experiments to investigate the liquidity of the phases in our kinetically regulated multiphase droplets. We used spot bleaching (r=0.8 μm) to measure the diffusivity of peptide 2, pSS, and pU in multiphase droplets with 50 mM fuel (Figure 4a and b). We spot‐bleached the pSS core and found that the diffusion of peptide 2 (0.007±0.003 μm^2^  s^−1^) is three to five times higher than the one of pSS which is expected based on the differences in molecular weight (Figure S11 and S12). Early in the cycle, the diffusion coefficient for both the peptide and pSS is low (Figure 4b) which implies that the droplet core is very dense right after fuel addition corresponding to a viscosity of 14.6 Pa⋅s; a value typical for dense coacervate droplets (see methods for calculation).^[33]^ Since the pU shell in multiphase droplets with 50 mM fuel is too thin for FRAP experiments, we performed spot bleaching on single‐phase pU droplets and assumed that their diffusivity is like the shell of the multiphase droplet (see supplementary discussion 2, Figure S13). The diffusion coefficient of peptide 2 in the pU phase is more than two orders of magnitude higher (4.0±2.5 μm^2^ s^−1^) compared to its diffusion in the pSS phase. Similarly, the pU diffuses four times faster in the shell compared to pSS in the core which is notable considering pU's much higher molecular weight (800 kDa vs 17 kDa). These differences in the diffusion coefficients imply that the core has a high viscosity, whereas the shell is more liquid. These findings were further corroborated by the fast fusion of the shell of the droplet (Movie S9 and S10), but the slow fusion of the core of the droplet (Figure 4c, Movie S10).

As the reaction cycle progressed, the diffusivity of the peptide and pSS increased drastically (Figure 4b, Figure S11 and S12) which was corroborated by fast fusion events of the pSS droplets (Figure 4c, Movie S11). At the time when the pU shell had dissolved and a vacuole has formed, the diffusivity of the peptide in the pSS phase, i.e., now the shell of the droplet, is almost two orders of magnitude higher compared to the diffusivity at the beginning of the cycle. We reason that as the levels of activated peptide 2 decrease, the droplets start to decay which leads to the efflux of molecules to give a more liquid compartment. Excitingly, we could also measure the diffusivity of the peptide in the vacuole, i.e., the newly formed pU core, and found it to be comparable to the diffusivity of the peptide in the pSS shell (Figure S14). Combined with the calculated concentrations inside the vacuole phase, we conclude that the vacuole phase is a liquid microenvironment, similar to the surrounding shell. In other words, the cycle starts with droplets with a very viscous pSS core surrounded by a liquid pU shell when fuel is abundant. These droplets transition to droplets with a liquid pU core surrounded by a liquid pSS shell when fuel is scarce (Figure 4d).

## Conclusion

Our work shows that active liquid droplets can display an inner hierarchy that is regulated through chemical reactions. The employed chemical reaction cycle controls the stability, liquidity, and location of the droplet multiphases. We show that phase transitions between phase‐separated states are unique to the active droplets and cannot be obtained in passive droplets under thermodynamic equilibrium. These active multiphase droplets bear similarities to their biological counterparts which often display complex architectures with liquid to solid‐like cores and shells. Our droplets also transition from multilayers with an RNA shell to multilayers with an RNA core^[58]^ which makes them a great model to elucidate mechanisms of organelle organization. Coacervate droplets have also been proposed as protocell models[^59^, ^60^, ^61^] and their regulation through chemical reactions might be thus relevant for the design of more complex compartments with metabolic networks.

## Experimental Section

Materials and methods description and additional data are available in the Supporting Information. The methods include: Synthetic protocols; Sample preparation; Isothermal titration calorimetry; UV/Vis spectroscopy; Fluorescence spectroscopy; Confocal fluorescence microscopy; FRAP procedures; Microfluidic procedures; HPLC procedures; Kinetic model and Calculations.

## Conflict of interest

The authors declare no conflict of interest.

1

## Supporting information

As a service to our authors and readers, this journal provides supporting information supplied by the authors. Such materials are peer reviewed and may be re‐organized for online delivery, but are not copy‐edited or typeset. Technical support issues arising from supporting information (other than missing files) should be addressed to the authors.

Supporting Information

Supporting Information

Supporting Information

Supporting Information

Supporting Information

Supporting Information

Supporting Information

Supporting Information

Supporting Information

Supporting Information

Supporting Information

Supporting Information

## Data Availability

The data that support the findings of this study are available from the corresponding author upon reasonable request.
