# Supplementary material for: Phase Transitions in Chemically Fueled, Multiphase Complex Coacervate Droplets
Source: Angew Chem Int Ed Engl. 2022 Oct 18;61(46):e202211905. doi: 10.1002/anie.202211905 (PMC9828839; doi:10.1002/anie.202211905)
Supplement: Supplementary file 1 — Supporting Information [file ANIE-61-0-s009.pdf]

Angewandte Chemie
Eine Zeitschrift der Gesellschaft Deutscher Chemiker

Supporting Information

Phase Transitions in Chemically Fueled, Multiphase Complex Coacervate Droplets

*C. Donau, F. Späth, M. Stasi, A. M. Bergmann, J. Boekhoven**

Materials and methods

Materials. We purchased 1-ethyl-3-(3-dimethylaminopropyl) carbodiimide (EDC), 2-(N-morpholino)ethanesulfonic acid (MES) buffer, trifluoroacetic acid (TFA), *N,N'*-diisopropylcarbodiimide (DIC), ethyl (hydroxyimino)cyanoacetate (Oxyma), 4-(dimethylamino)-pyridine (DMAP), Wang resin, Rinkamide resin, protected amino acids (Fmoc-N(Trt)-OH, Fmoc-D(OtBu)-OH, Fmoc-G-OH, Fmoc-R(Pbf)-OH, Ac-F-OH), Wang resin, piperidine (99%), triisopropylsilane (TIPS), chloro-7-nitrobenzofurazan (NBD-Cl, 98%), polyuridylic acid potassium salt (pU), poly(styrene sulfonate) sodium salt (pSS), 2-(Dodecylthiocarbonothioylthio)-2-methylpropionic acid (98%), 4,4'-Azobis(4-cyanovaleric acid (ACPA, ≥98%), sodium borohydride, tris(2-carboxyethyl) phosphine (TCEP), ethylenediamine, sodium 4-vinylbenzenesulfonate (SVBS), sulforhodamine 101 and solvents (*N,N*-dimethylformamide (DMF), dichloromethane, diethyl ether) were purchased from Sigma-Aldrich and used without any further purification unless otherwise indicated. Sulfo-cyanine5-maleimide(1-(6-((2-(2,5-dioxo-2,5-dihydro-1H-pyrrol-1-yl)ethyl)amino)-6-oxohexyl)-3,3-dimethyl-2-((1E,3E)-5-((E)-1,3,3-trimethyl-5-sulfonatoindolin-2-yldene)-penta-1,3-dien-1-yl)-3H-indol-1-ium-5-sulfonate) was purchased from Lumiprobe. Nuclease-free water was freshly prepared by filtration (DURAN) of MQ-water. HPLC grade acetonitrile (ACN) was purchased from VWR. Cy3-A_{15}, Cy5-A_{15}, and Cy5-U_{15} were purchased from biomers.net GmbH. Ac-FRGRGRGD-OH (tri-trifluoroacetic acid salt) was purchased from CASLO Aps. Ac-FRGRGRGN-NH_2 (tri-trifluoroacetic acid salt) was manually synthesized (see below) and purchased from CASLO Aps. NBD-GRGRGRGN-NH_2 and NBD-GRGRGRGD-OH (both as tri-trifluoroacetic acid salt) were synthesized following a published procedure[2]. Cy5-pSS was synthesized using RAFT polymerization (see below).

Methods

Synthesis of Ac-FRGRGRGN-NH_2. The peptide was synthesized using standard fluoren-9-ylmethoxycarbonyl (Fmoc) solid-phase peptide synthesis on Rinkamide resin on a CEM Liberty microwave-assisted peptide synthesizer. The purity of the peptide was analyzed by electrospray ionization mass spectrometry in positive mode (ESI-MS) and analytical HPLC.

Synthesis of Cy5-pSS. Poly(styrene sulfonate) homopolymer was synthesized via RAFT polymerization. For this purpose, styrene sulfonate sodium salt (SVBS) was polymerized in MQ-water at 75°C with 0.01 eq. 2-(Dodecylthiocarbonothioylthio)-2-methylpropionic acid (98%) and 0.002 eq. of 4,4'-Azobis(4-cyanovaleric acid (ACPA, ≥98%) for 5 hours. The solution was purged before with Argon for 20 minutes to remove air. The crude solution was purified by dialysis against MQ-water in 12-14 kDa MWCO dialysis tubings (Spectra/Por© 4, 29 mm diameter) for 2 days. The product was dried by lyophilization. The CTA-terminated poly(styrene sulfonate) (440 mg) was solubilized in 15 mL MQ-water, 15 mL of a freshly prepared 1M $NaBH_4$ solution were added dropwise and the mixture was then

stirred for 3.5 hours at room temperature. The crude solution was dialyzed against MQ-water for 2 days in 12-14 kDa MWCO dialysis tubings (Spectra/Por© 4, 29 mm diameter) and lyophilized. The resulting product was then reacted with 150 eq. of tris(2-carboxyethyl) phosphine (TCEP) at room temperature in DMF for 24 hours. 20 eq. of Sulfo-Cyanine5-maleimide(1-(6-((2-(2,5-dioxo-2,5-dihydro-1H-pyrrol-1-yl)ethyl)amino)-6-oxohexyl)-3,3-dimethyl-2-((1E,3E)-5-((E)-1,3,3-trimethyl-5-sulfonatoindolin-2-ylidene)penta-1,3-dien-1-yl)-3H-indol-1-ium-5-sulfonate) were added to an aliquot of this solution together with a catalytic amount of ethylenediamine and stirred at 50 °C for 24 hours. The crude product was purified by dialysis in a 2000 MWCO Slide-A-Lyzer Dialysis Cassette© (ThermoFisher Scientific) against MQ-water for 2 days and finally lyophilized. Analysis from SEC (Figure S16): Mw = 710 kDa, PDI = 1.4.

Sample preparation. Stock solutions of peptide 2 (Ac-FRGRGRGD-OH, 100-150 mM), MES buffer (500 mM), pU (600-1000 kDa, 15 µg/µL), pSS (17 kDa, 166 mg/mL), fluorescent dyes (1-25 µM), peptide 1 (Ac-FRGRGRGN-NH_2, 1-75 mM) and fuel (EDC, 0.5-2 M) were prepared in nuclease-free water. The pH of the peptide and MES stock were adjusted to 5.3. Typically, stock solutions of EDC, as well as the resulting peptide-polymer solution in MES, were prepared freshly for each experiment. For most experiments, we used these standard conditions for active droplets: 15 mM peptide 2, 5 mM pU (monomer units), 5 mM pSS (monomer units), 200 mM MES, pH 5.3, and 50 mM fuel. Reaction networks were started by the addition of fuel to the peptide-polymer solution in MES. For passive droplets, we used these standard conditions: x mM peptide 1, 15-x mM peptide 2, 5 mM pU, 5 mM pSS, 200 mM MES, pH 5.3. Droplet formation was initiated by the addition of a mixture of pSS and pU (with dyes) to a solution in MES containing both peptide 1 and peptide 2.

Hybridization of pU. To track pU fluorescently, we hybridized pU with Cy3-A_{15} or Cy5-A_{15}. For that, pU and the fluorescent A_{15} were added into an Eppendorf tube (5 µL). The mixture was incubated at 80 °C for 5 minutes following 0 °C for 5 minutes. The resulting solution was then stored at 25 °C and consumed within an hour.

Isothermal titration calorimetry (ITC). ITC Experiments were performed with a MicroCal PEAQ-ITC from Malvern Pananalytical. All experiments were performed at 25°C. The following conditions were used: pU (1.1 mM uridine units in MES 200 mM, pH 5.3) was titrated with peptide 2 (38 mM in 200mM MES, pH 5.3): 25 injections, 1.5 uL/inj. pU (0.5 mM in 200 mM MES, pH 5.3) was titrated with peptide 1 (5 mM as charged units, 1.67 mM as peptide concentrations in 200 mM MES, pH 5.3): 19 injections, 2uL/inj. pSS (1 mM sulfonate unit in 200 mM MES, pH 5.3) was titrated with peptide 2 (15 mM in 200 mM MES, pH 5.3): 25 injections, 1 uL/inj. pSS (0.025 mM sulfonate unit in 200 mM MES, pH 5.3) was titrated with peptide 1 (0.375 mM as charged units, 0.125 mM as peptide concentrations in 200 mM MES, pH 5.3): 16 injections, 2uL/inj. All experiments were performed in triplicate (N=3). For each

experiment, a control was performed by titrating the corresponding amount of peptide in 200 mM MES buffer (pH 5.3) in absence of polyanions. Data were fitted using a non-linear least squares algorithm provided with the PEAQ-ITC Analysis software. All titrations were performed in a regime of concentrations where no phase-separation behavior could be observed.

UV/Vis Spectroscopy. The UV/Vis measurements were carried out using a Microplate Spectrophotometer (Thermo Scientific Multiskan GO). Samples (100 µL) were directly prepared into a 96 well-plate (tissue culture plate non-treated) and the absorbance as a measure for turbidity was monitored at 600 nm every minute. Measurements were performed at 25 °C. Each experiment was performed in triplicate.

Fluorescence Spectroscopy. Fluorescence spectroscopy was performed on a Jasco (Jasco FP-8300) spectrofluorimeter. Samples (100 µL) were added to a cuvette and the fluorescence was measured at specific wavelengths depending on the dye (see confocal settings). Measurements were performed at 24°C. Each experiment was performed in triplicate.

Confocal Fluorescence Microscopy. Confocal fluorescence microscopy was performed on a Leica TCS SP8 confocal microscope using a 63x water immersion objective (1.2 NA). Samples (V = 30 µL) were prepared as described above in PVA-coated[2] micro-well plates (ibidi, µ-Slide Angiogenesis Glass Bottom), but with 0.15 µM Cy3-RNA, 0.15 µM Cy5-RNA, 0.15 µM Cy5-pSS and/or 1 µM NBD-peptide as the fluorescent dye. Samples were excited with 488 nm (NBD), 552 nm (Cy3), and 638 nm (Cy5) and imaged at 498-550 nm, 560-630 nm, and 650-710 nm, respectively. A sequential scan was employed when more than one dye was present in the sample. Images of passive droplets were typically obtained 10 minutes after droplet formation. Measurements were performed at 24 °C.

Fluorescent recovery after photobleaching (FRAP). The diffusivity of the molecules inside active droplets was measured via spot bleaching. The region of interest (ROI) was set to a radius of 0.8 µm inside droplets with a diameter of a couple of micrometers. For peptide 2, samples containing 1 µM NBD-GRGRGRGD-OH (fluorescent peptide 2) at standard conditions were bleached and excited with a 488 nm laser and imaged at 498-550 nm with an image size of 144x80 pixels. 5 pre-bleaches (every 53 ms) were acquired followed by 3 bleaching pulses (every 53 ms). After that, fluorescence recovery was tracked every 53-500 milliseconds with a total of up to 107 images. For pSS, samples containing 0.15 µM Cy5-pSS at standard conditions were bleached and exited at 638 nm and imaged at 650-710 nm with an image size of 256x100 pixels. 5 pre-bleaches (every 0.19 s) were acquired followed by 10 bleaching pulses (every 0.19 s). After that, fluorescence recovery was tracked every 0.19-1.0 seconds with a total of up to 163 images. To measure the diffusivity of pSS and pU in samples with either 10

mM pSS or pU at standard conditions with 0.15 µM dye, samples were bleached and exited at either 552 nm or 638 nm and imaged at 560-630 or 650-710 nm with an image size of 512x64 pixels. The raw data was then grouped into time regimes after fuel addition (*e.g.* 0-7 min, 10-15 min,..). After double normalization[3], the data were fitted to a first-order exponential equation[4] with the spot size and the diffusion coefficient to obtain the diffusion coefficient and half-time recovery, as previously described.[2] For the vacuole phase, it was only possible to immobilize the vacuole for a couple of seconds. As a result, we could only accurately determine the diffusion of the peptide in the vacuole since it is a small molecule, and its fluorescent intensity recovers much faster than pU and pSS. Here, the image size was 80x40 pixels and the raw data was only subtracted from the background without normalization of neighboring droplets. To measure the diffusivity of pU in the pSS phase of active multiphase droplets, we hybridized pU with Cy5-A_{15}-pU in presence of sulforhodamine (to better visualize the droplets). Samples were then bleached and excited with a 638 nm laser and imaged at 650-710 nm with an image size of 144x80 pixels. 5 pre-bleaches (every 53 ms) were acquired followed by 10 bleaching pulses (every 53 ms). After that, fluorescence recovery was tracked every 53-500 milliseconds with a total of up to 107 images. Since the partitioning of pU in the pSS phase is close to 1, we did not subtract the background but only corrected it for photobleaching by a neighboring droplet. Error bars for all experiments show the standard deviation from two experiments with three droplets each (*N=6*) per time point.

Microfluidic chip production:
Microfluidic PDMS (Polydimethylsiloxane, Sylgard 184, Dow Corining)-based devices were designed with QCAD-pro (RibbonSoft GmbH) and fabricated using photo- and soft-lithography[5] as previously described.[6]

Microfluidic droplet formation:
Surfactant-stabilized water in oil droplets was produced using 2% 008-FluoroSurfactant in 3M Novec7500 as the oil phase, fuel in MQ water as one of the water phases and peptide 2, polyanions and dyes in MES buffer at pH 5.3 as the second water phase. Both water phases contained the doubled concentrations of the respective components with respect to the standard conditions and were mixed in a 1-to-1 ratio to yield the standard concentrations inside the microfluidic droplets. Both aqueous phases and the oil phase were injected into the microfluidic PDMS-based device through polytetrafluoroethylene (PTFE) tubes (0.4-0.9 mm, Bola, Germany) using a flow control system (ELVEFLOW Pressure Controller OB1 MK3). Typically, pressures of 610 mbar for the water phases and 810 mbar for the oil phase were used to produce stable water in oil droplets with a diameter of 40 µm.

HPLC. The concentration profiles of the fuel, peptide 2, and activated peptide 2 during the chemical reaction cycle were monitored over time using analytical HPLC (ThermoFisher,

Vanquish Duo UHPLC, HPLC) with a Hypersil Gold 100 2.1 mm C18 column (3 mm pore size). To determine the EDC concentration over time, a turbid sample (V = 10 µL) of active droplets in an HPLC vial with an inlet at standard conditions was diluted with 10 µL of an aqueous NaCl solution (4M) at a given time point and directly injected into the HPLC. Separation was performed using a linear gradient of ACN (2 to 98%) and water with 0.1% TFA and the chromatogram was analyzed using detectors at 220 nm and 254 nm. To determine the activated peptide 2 concentration, a benzylamine quench was used.[1b, 7] Briefly, at each time point, 10 µL from a sample containing active droplets (V = 120 µL) in a 96 well plate were added into 20 µL of an aqueous solution of benzylamine (300 mM) in an HPLC vial. After the addition of 10 µL of an aqueous NaCl solution (4M), the samples were directly injected into the HPLC with the settings from above. All measurements were performed at 25 °C. Each experiment was performed in triplicate.

Kinetic model. A kinetic model written in MATLAB was used to predict the evolution of fuel, peptide 2, and activated peptide 2 over time. The model is described in detail in our previous work.[1] The MATLAB code is available at https://github.com/BoekhovenLab/Dynamic-droplets.

Measuring the concentrations in the phase outside of the droplets ($c_{outside}$). Passive droplets: a solution of passive droplets (V = 150 µL) prepared at standard conditions with 1 µM NBD-GRGRGRGN-NH_2 (fluorescent peptide 1) and 0.15 µM Cy5-pSS in an Eppendorf tube was vortexed for 10 seconds and then centrifuged for 10 minutes at 20,412×g. The supernatant (V = 100 µL) was removed and added to an Eppendorf tube containing 1 µL of an aqueous solution of NaCl (4 M) to dissolve residual turbidity. The fluorescence of the sample was then measured on the fluorimeter. To account for the dependence of the fluorescence of dyes on their environment,[8] we prepared an identical sample w/o dye and added the supernatant (85-95 µL) to an Eppendorf tube containing 1 µL of an aqueous solution of NaCl (4 M). To the clear solution, we then added the same amount of dye (5-15 µL) as in the previous sample and measured its fluorescence intensity. The intensity ratio between these 2 samples was therefore the fraction of the fluorescent molecules that remained in the supernatant. Error bars show the standard deviation from the average (*N=3*).
Active droplets: to a solution of active droplets (V = 150 µL) prepared at standard conditions with 1 µM NBD-GRGRGRGD-OH (fluorescent peptide 2), 0.15 µM Cy3-A_{15} or 0.15 µM Cy5-pSS, 50 mM EDC was added. At a given timepoint, the sample was centrifuged for 1 min at 20,412×g. The fraction of fluorescent molecules that remained in the supernatant was then measured and quantified as for passive droplets. Since fluorescent peptide 2 can also be activated by the fuel, the measured intensity is reflecting the partitioning of peptide 2 and activated peptide 2 combined. Error bars show the standard deviation from the average (*N=3*).

Calculating the concentrations inside the droplets and the resulting partitioning coefficient. We determined the droplet volumes of passive droplets at standard conditions after centrifugation of the turbid suspensions (V = 150 µL) for 10 minutes at 20,412×g. The resulting droplet pellet (0.2 – 1.5 µL) was then compared to size standards visually. We then calculated the concentration in the droplet phase by subtracting the number of molecules in the phase outside of the droplets from the total amount in the solution. Partitioning coefficients K were obtained by the ratio of the concentration in the droplets and the concentration outside of the droplets.

Calculating concentrations inside the vacuole phase. We measured the fluorescence intensity profiles of active droplets via confocal fluorescence microscopy, 22 minutes after the addition of 50 mM fuel under standard conditions. The resulting partitioning coefficients $K_{vacuole}$ were then multiplied with the concentration in the phase outside of the droplets $c_{outside}$ obtained from the fluorimeter (see method above) to give the concentrations inside the vacuole $c_{vacuole}$ (Table S2). We performed control experiments to verify that the dye's fluorescence hardly varies with the pSS concentration. Specifically, we prepared individual samples with identical dye concentrations in 200 mM MES at pH 5.3 and varied the pSS concentration up to 30 mM. In the regime between 4 mM and 30 mM pSS, the increase of the dyes' fluorescence was negligible. Error bars of $K_{vacuole}$ show the standard deviation from 5 droplets containing vacuoles (*N = 5*). Error bars of $c_{vacuole}$ were derived from error bars of $c_{outside}$ and $K_{vacuole}$.

Estimating the viscosity of the pSS phase. To estimate the viscosities of the pSS phase during the reaction cycle, we used the Stokes-Einstein relationship: $D = \frac{k_B T}{6\pi\eta R_h}$, where D is the diffusion coefficient, k_B is the Boltzmann constant, T is the temperature, η is the viscosity and R_h is the hydrodynamic radius. Under the assumption of the droplets behaving as equilibrium Newtonian liquids, the droplets' viscosity can be estimated as a first approximation.[9] D values of pSS were used from FRAP experiments. R_h of pSS was assumed to be approximately 10 nm based on the literature.[10]

Determining the critical coacervation concentration (CCC) and critical salt concentration (CSC) of passive droplets. The CCC concentrations of passive droplets were determined by increasing the amount of peptide 1 gradually by pipetting increasing amounts of a 100 mM stock solution into a sample containing either 10 mM pSS or 10 mM pU in 200 mM MES at pH 5.3. Turbidity was measured by UV/vis spectrometry at 600 nm. Turbidity values above the blank value (0.085 absorbance) were taken as the indicator for phase separation.

Critical salt concentrations were determined accordingly, but different amounts of a 4M NaCl stock solution were added to samples containing 2.5 mM of peptide 1, 10 mM of pSS or 10 mM pU in 200 mM MES at pH 5.3.

Supplementary discussion 1:

We performed a control experiment to indirectly demonstrate that A_{15} must be mostly hybridized even in the droplet environment. For that, we used our conditions for active multiphase droplets based on pU and pSS. As dyes, we used Cy3-A_{15}-pU and Cy5-U_{15} instead of Cy5-pSS. Cy5-U_{15} cannot base-pair with pU. Indeed, we observed that U15 localized into the pSS phase during vacuole formation (Figure S7). Thus, there is a significant difference in the partitioning of non-hybridized U_{15} compared to hybridized A_{15}. A_{15} and U_{15} should only show disparate partitioning in multiphase droplets if base-pairing is present,[11] i.e., the pU-phase selects for one of the two nucleic acids via hybridization. Thus, we conclude that the Cy3-emission from A_{15} indicates pU's location.

Supplementary discussion 2:

The pU shell in active multiphase droplets was too thin for FRAP spot bleaching experiments. Instead, we performed FRAP on pU-only droplets (10 mM) as an indirect measure for the pU shell (Figure S13). To verify whether this assumption was true, we performed control experiments on pSS-only droplets (Figure S15). Here, the diffusivities obtained for the peptide and pSS were in the same range as for the pSS core in multiphase droplets, indicating that the measured diffusivities in pU-only droplets can be used as an estimate for the pU shell in multiphase droplets.

Table S1. *Critical coacervation concentration (CCC) and critical salt concentration (CSC) of passive pSS and pU droplets with peptide 1.*

	**pSS**	**pU**
CCC	0.8 ± 0.1 mM	1.85 ± 0.05 mM
CSC	>1.5 M	63 ± 12 mM

Values derived from UV-vis spectroscopy. Conditions for CCC of peptide 1: 10 mM pSS or 10 mM pU, 200 mM MES, pH5.3. Conditions for CSC of NaCl: 2.5 mM peptide 1, 10 mM pSS or 10 mM pU, 200 mM MES, pH5.3. Error bars show the standard deviation from the average (N=3).

Table S2. *Concentrations of molecules in the vacuole phase of active droplets*.

	**pSS**	**pU**	**Peptide 2***
$c_{outside}$	4.0 ± 0.2 mM	5.1 ± 0.1 mM	8.4 ± 0.4 mM
$K_{vacuole}$	4.7 ± 1.3	2.2 ± 0.1	10.6 ± 0.3
$c_{vacuole}$	18.8 ± 6.2 mM	11.2 ± 0.7 mM	89.0 ± 6.3 mM

**Concentrations represent peptide 2 and activated peptide 2 combined.*

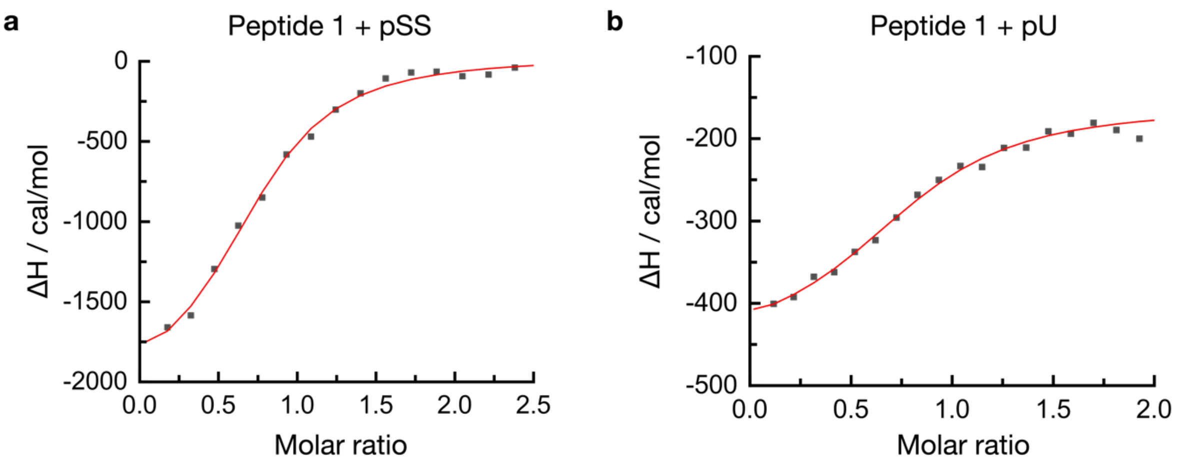

c

K_D	pSS	pU
Peptide 1	3.2 ± 1.5 μM	130 ± 50 μM
Peptide 2	110 ± 1.0 μM	14 ± 4.5 mM

d

**ΔG / kcal mol^{-1}**	pSS	pU
Peptide 1	-7.55 ± 0.3	-5.33 ± 0.3
Peptide 2	-5.42 ± 0.0	-2.60 ± 0.3

Figure S1: *Binding affinity between the peptides and polyanions. (**a-b)** Change in enthalpy measured by ITC for the interaction between peptide 1 and pSS (a) and pU (b). (**c-d)** Dissociation constant (c) and free energy (d) of the interaction between peptide 1 and 2 with the two polyanions. Errors show the standard deviation from the average (N=3). For conditions, see methods.*

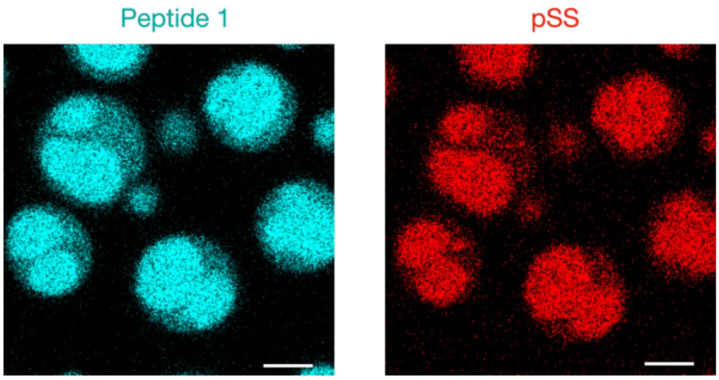

Figure S2: *Localization of peptide 1 in passive multiphase droplets. Confocal micrographs of fluorescent peptide 1 (left) and pSS (right) of passive droplets. Conditions: 2.5 mM peptide 1, 12.5 mM peptide 2, 5 mM pU, 5 mM pSS, 200 mM MES, pH5.3, 1 μM NBD-GRGRGRGN-NH_2 (cyan) and 0.15 μM Cy5-pSS (red). Scale bar: 3 μm.*

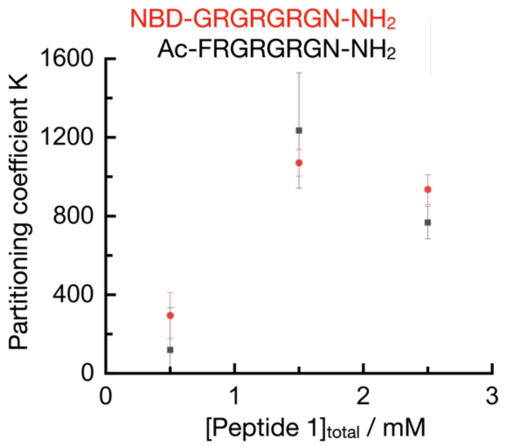

Figure S3: *Partitioning of peptide 1 in passive droplets. Partitioning coefficient of NBD-GRGRGRGN-NH_2 (fluorescent peptide 1, red) and Ac-FRGRGRGN-NH_2 (peptide 1, black) in the droplet phase as a function of the total concentration of peptide 1 for passive droplets at standard conditions. Values of the unlabeled peptide were determined by centrifugation and HPLC. Error bars show the standard deviation from the average (N=3).*

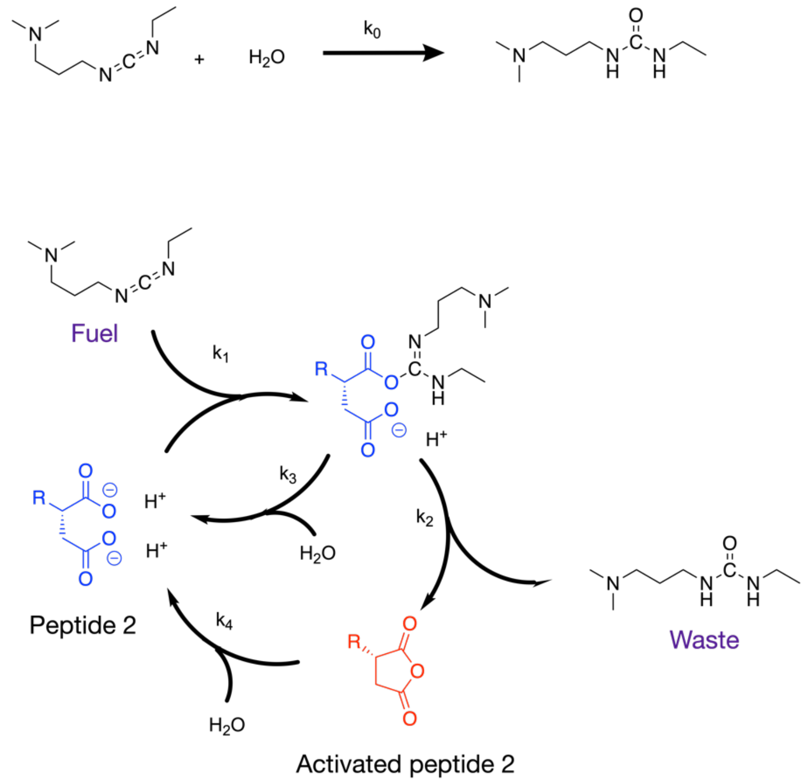

Figure S4: *Chemical reaction cycle in active droplets. All reactions taking place simultaneously. This reaction cycle has been described in detail in our previous work.[1] The rate constants (k) refer to the rate constants used in our kinetic model.*

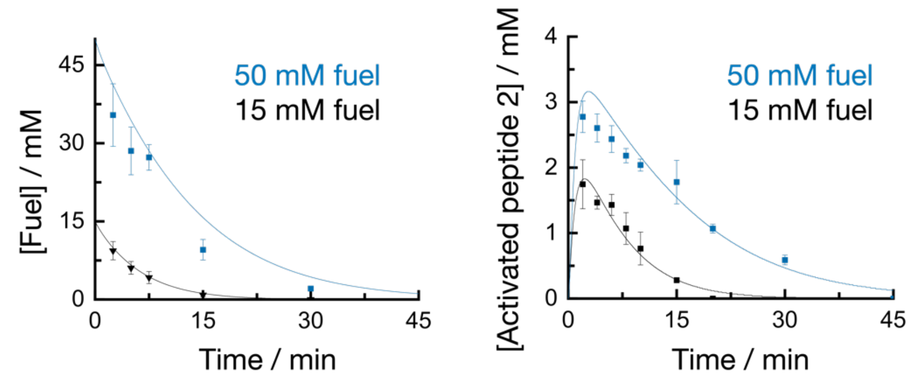

Figure S5: *Evolution of fuel and peptide concentrations over time. Fuel and peptide concentrations over time measured by HPLC for 15 mM (black) and 50 mM (blue) fuel at standard conditions for active droplets. Error bars show the standard deviation from the average (N=3). Lines represent fitting plots from our kinetic model. Fitting parameters:* $k_1(15mM) = 0.1\ M^{-1}s^{-1}$, $k_1(50mM) = 0.05\ M^{-1}s^{-1}$, $k_2 = 0.2\ s^{-1}$, $k_3 = 0\ s^{-1}$, $k_4 = 0.015\ s^{-1}$.

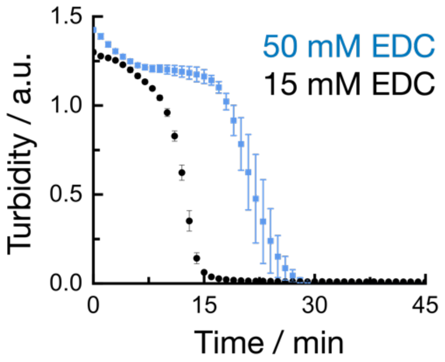

Figure S6: *Evolution of turbidity in active droplets over time. Absorbance of 600 nm of light as a measure for turbidity for standard conditions of active droplets with 15 mM (black) or 50 mM (blue) fuel. Error bars show the standard deviation from the average (N=3).*

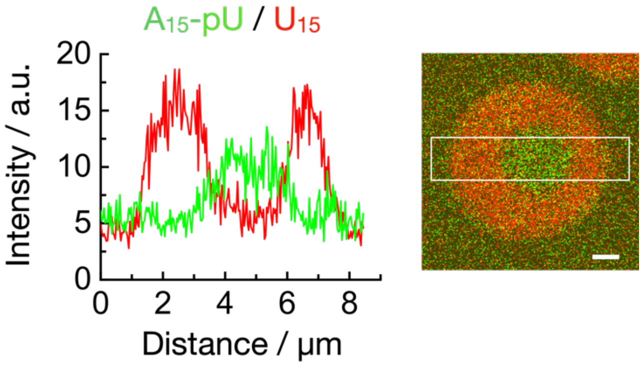

Figure S7: *Comparing the partitioning of A_{15} and U_{15} in active droplets at transition 3. Confocal micrographs of a sample containing active droplets at standard conditions, 22 minutes after the addition of 50 mM fuel but with Cy3-A_{15}-pU (green) and Cy5-U_{15} (red) instead of Cy5-pSS. Scale bar: 1 µm.*

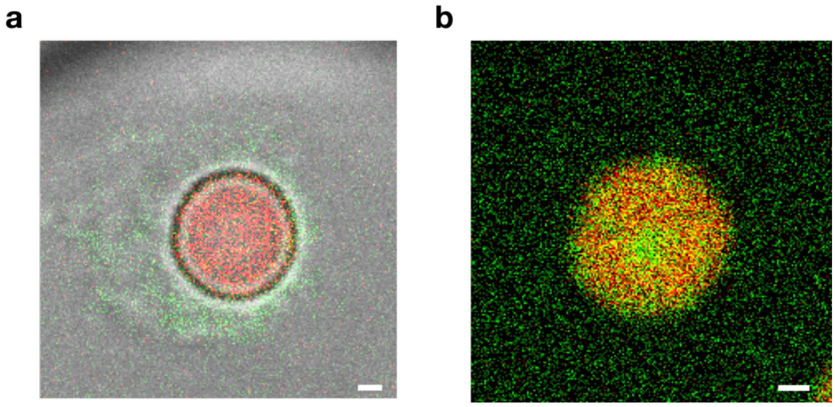

Figure S8*: Transition 2 and 3 in the microfluidic set-up. (a) Dissolution of pU at standard conditions of active droplets with 50 mM fuel, 0.15 µM Cy3-A15-pU (green) and 0.15 µM Cy5-pSS (red). (b) Re-partitioning of pU into the pSS phase. Scale bar: 1 µm.*

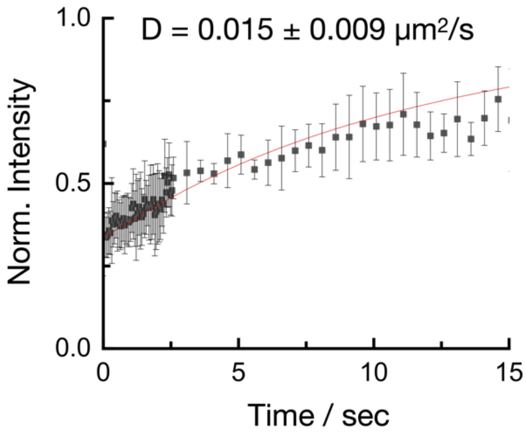

Figure S9: *Diffusivity of pU in the pSS phase in active multiphase droplets. FRAP spot bleaching experiments of active droplets with 50 mM fuel at standard conditions with 0.15 µM Cy5-A_{15}-pU and 0.15 µM sulforhodamine at various time points. Red lines indicate fitting functions with corresponding diffusion coefficient D. Errors bars show the standard deviation of 6 droplets from 2 experiments (N=6).*

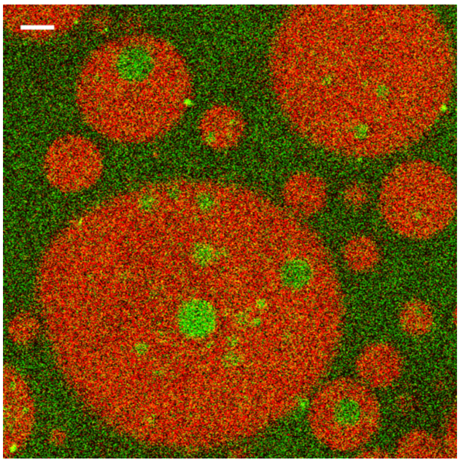

Figure S10: *Vacuole conditions in the bulk phase. Confocal micrographs of fluorescent pU (green) and pSS (red) of a sample containing similar concentrations of molecules as the vacuole phase in active droplets. Conditions: 89 mM peptide 2, 11 mM pU, 19 mM pSS, 200 mM MES, pH5.3, 0.15 µM Cy3-A_{15}-pU (green) and 0.15 µM Cy5-pSS (red). Scale bar: 5 µm.*

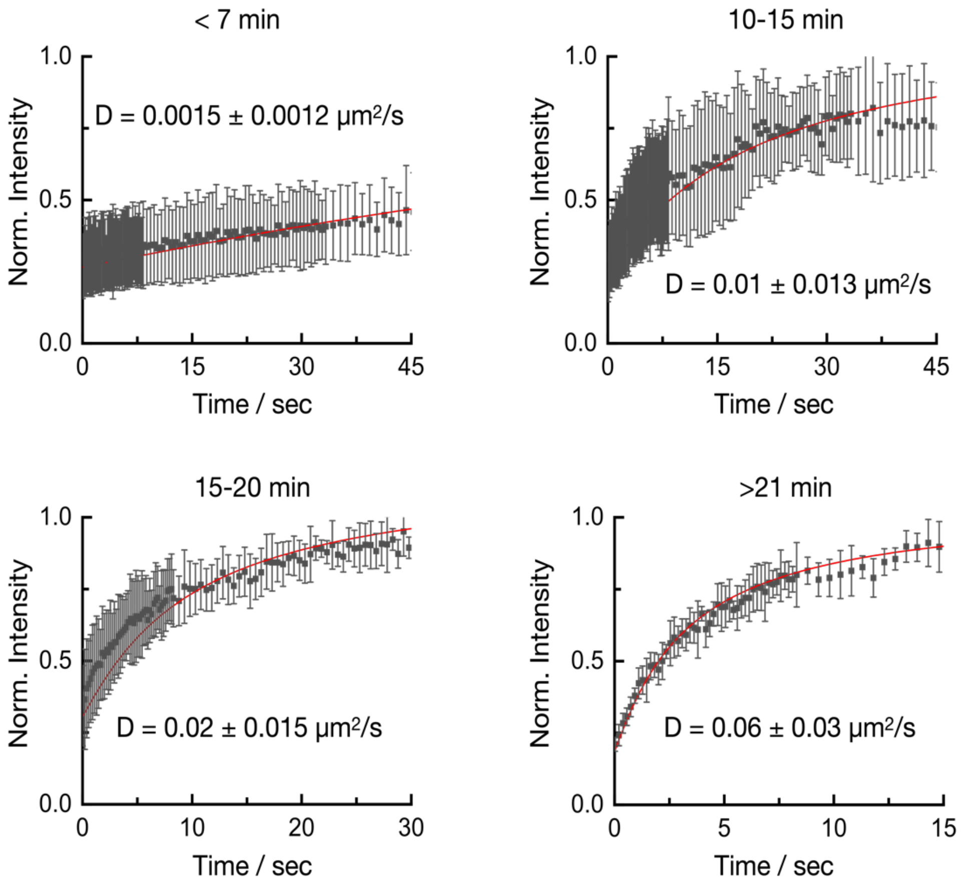

Figure S11: *Diffusivity of pSS in the pSS phase in active multiphase droplets. FRAP spot bleaching experiments of active droplets with 50 mM fuel at standard conditions with 0.15 µM Cy5-pSS at various time points. Red lines indicate fitting functions with corresponding diffusion coefficient D. Errors bars show the standard deviation of 6 droplets from 2 experiments (N=6).*

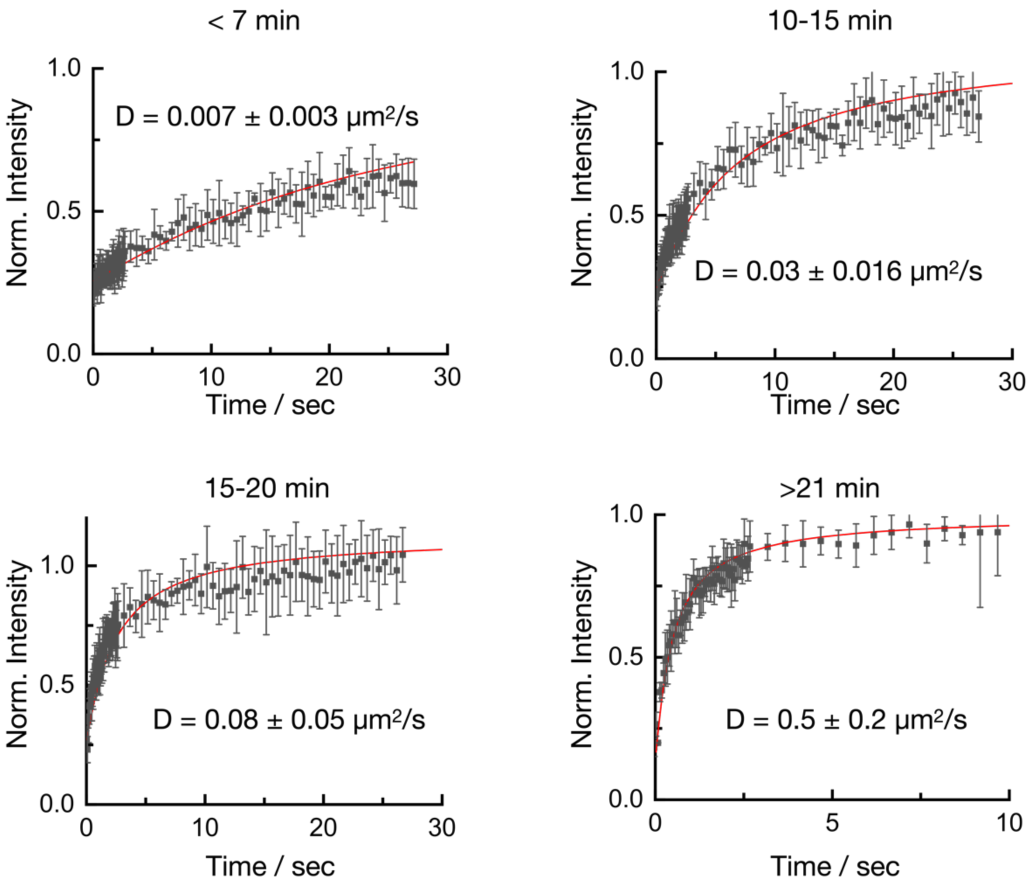

Figure S12: *Diffusivity of the peptide in the pSS phase in active multiphase droplets. FRAP spot bleaching experiments of active droplets with 50 mM fuel at standard conditions with 1 µM NBD-GRGRGRGD-OH at various time points. Red lines indicate fitting functions with corresponding diffusion coefficient D. Errors bars show the standard deviation of 6 droplets from 2 experiments (N=6). Since the fluorescent peptide can react with the fuel, the measured recovery represents both peptide 2 and activated peptide 2.*

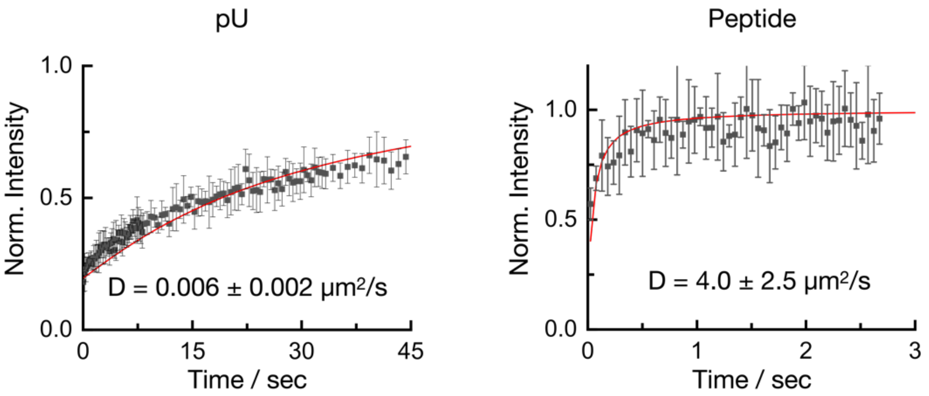

Figure S13: *Diffusivity of pU and the peptide in pU-only active droplets. FRAP spot bleaching experiments of pU-only droplets with 50 mM fuel at standard conditions (10 mM pU w/o pSS) with 0.15 µM Cy3-A_{15}-pU or 1 µM NBD-GRGRGRGD-OH during the first minutes after fuel addition. Red lines indicate fitting functions with corresponding diffusion coefficient D. Errors bars show the standard deviation of 6 droplets from 2 experiments (N=6). Since the fluorescent peptide can react with the fuel, the measured recovery represents both peptide 2 and activated peptide 2.*

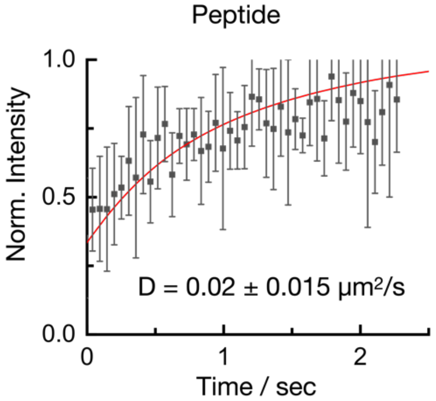

Figure S14: *Diffusivity of the peptide in the vacuole phase in active multiphase droplets. FRAP spot bleaching experiments of active droplets with 50 mM fuel at standard conditions with 1 µM NBD-GRGRGRGD-OH in the vacuole phase. Red lines indicate fitting functions with corresponding diffusion coefficient D. Errors bars show the standard deviation of 6 droplets from 2 experiments (N=6). Since the fluorescent peptide can react with the fuel, the measured recovery represents both peptide 2 and activated peptide 2.*

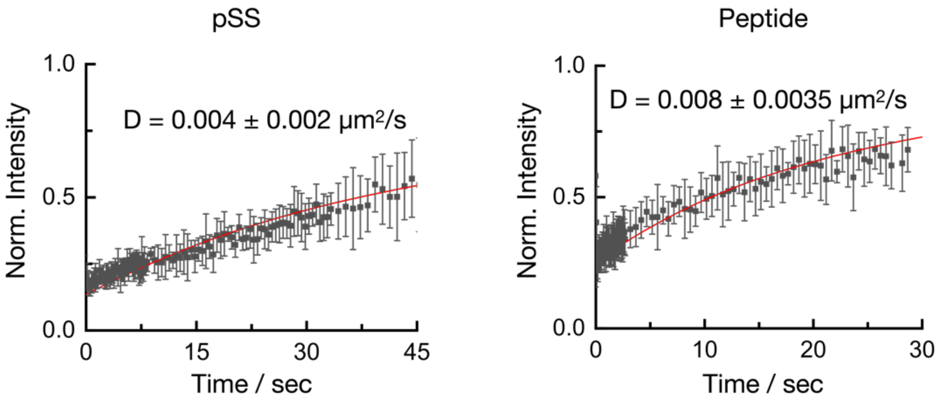

Figure S15: *Diffusivity of pSS and the peptide in pSS-only active droplets. FRAP spot bleaching experiments of pSS-only droplets with 50 mM fuel at standard conditions (10 mM pSS w/o pU) with 0.15 µM Cy5-pSS or 1 µM NBD-GRGRGRGD-OH during the first minutes after fuel addition. Red lines indicate fitting functions with corresponding diffusion coefficient D. Errors bars show the standard deviation of 6 droplets from 2 experiments (N=6). Since the fluorescent peptide can react with the fuel, the measured recovery represents both peptide 2 and activated peptide 2.*

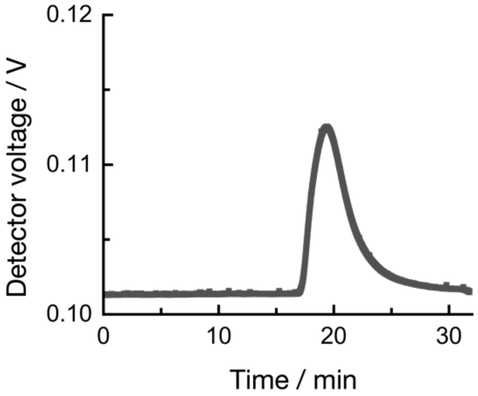

Figure S16*: Characterisation of the synthesized Cy5-pSS. Size Exclusion Chromatography (SEC) chromatogram of unmodified poly(styrene sulfonate) in a 1:1-mixture of H_2O and THF with 9 g/L tetra-n-butylammonium bromide (TBAB) and 340 mg/L 3,5-di-tert-butyl-4-hydroxytoluene (BHT) as the eluent at 40 °C and a flow rate of 0.6 mL/min. A combination of two Agilent PL PolargelM columns (7.5 x 300 mm) was used.*

References

[1] a) M. Tena-Solsona, B. Riess, R. K. Grotsch, F. C. Lohrer, C. Wanzke, B. Kasdorf, A. R. Bausch, P. Muller-Buschbaum, O. Lieleg, J. Boekhoven, *Nat. Commun.* **2017**, *8*, 15895; b) C. Donau, F. Spath, M. Sosson, B. A. K. Kriebisch, F. Schnitter, M. Tena-Solsona, H. S. Kang, E. Salibi, M. Sattler, H. Mutschler, J. Boekhoven, *Nat. Commun.* **2020**, *11*, 5167.

[2] F. Spath, C. Donau, A. M. Bergmann, M. Kranzlein, C. V. Synatschke, B. Rieger, J. Boekhoven, *J. Am. Chem. Soc.* **2021**, *143*, 4782-4789.

[3] S. A. G. Robert D. Phair, Tom Misteli, *Meth. Enzymol.* **2003**, *375*, 393-414.

[4] A. B. Kayitmazer, H. B. Bohidar, K. W. Mattison, A. Bose, J. Sarkar, A. Hashidzume, P. S. Russo, W. Jaeger, P. L. Dubin, *Soft Matter* **2007**, *3*, 1064-1076.

[5] J. C. M. David C. Duffy, Olivier J. A. Schueller, and George M. Whitesides, *Anal. Chem.* **1998**, *70*, 4974–4984.

[6] a) M. Weiss, J. P. Frohnmayer, L. T. Benk, B. Haller, J. W. Janiesch, T. Heitkamp, M. Borsch, R. B. Lira, R. Dimova, R. Lipowsky, E. Bodenschatz, J. C. Baret, T. Vidakovic-Koch, K. Sundmacher, I. Platzman, J. P. Spatz, *Nat. Mater.* **2018**, *17*, 89-96; b) T. W. Hofmann, S. Hanselmann, J. W. Janiesch, A. Rademacher, C. H. Bohm, *Lab Chip* **2012**, *12*, 916-922.

[7] F. Schnitter, J. Boekhoven, *ChemSystemsChem* **2020**, *3*, e2000037.

[8] T. J. Nott, T. D. Craggs, A. J. Baldwin, *Nat. Chem.* **2016**, *8*, 569-575.

[9] a) C. P. Brangwynne, C. R. Eckmann, D. S. Courson, A. Rybarska, C. Hoege, J. Gharakhani, F. Julicher, A. A. Hyman, *Science* **2009**, *324*, 1729-1732; b) B. Drobot, J. M. Iglesias-Artola, K. Le Vay, V. Mayr, M. Kar, M. Kreysing, H. Mutschler, T. D. Tang, *Nat. Commun.* **2018**, *9*, 3643.

[10] U. Böhme, U. Scheler, *Macromol. Chem. Phys.* **2007**, *208*, 2254-2257.

[11] G. A. Mountain, C. D. Keating, *Biomacromolecules* **2020**, *21*, 630-640.
